# Supplementary material for: A Phylogeny of Birds Based on Over 1,500 Loci Collected by Target Enrichment and High-Throughput Sequencing
Source: PLoS One. 2013 Jan 29;8(1):e54848. doi: 10.1371/journal.pone.0054848 (PMC3558522; doi:10.1371/journal.pone.0054848)
Supplement: Figure S1 — Fully resolved trees from the 1,541 locus analysis with support values. A. Bayesian tree. B. Maximum-likelihood tree. (DOCX) [file pone.0054848.s001.docx]

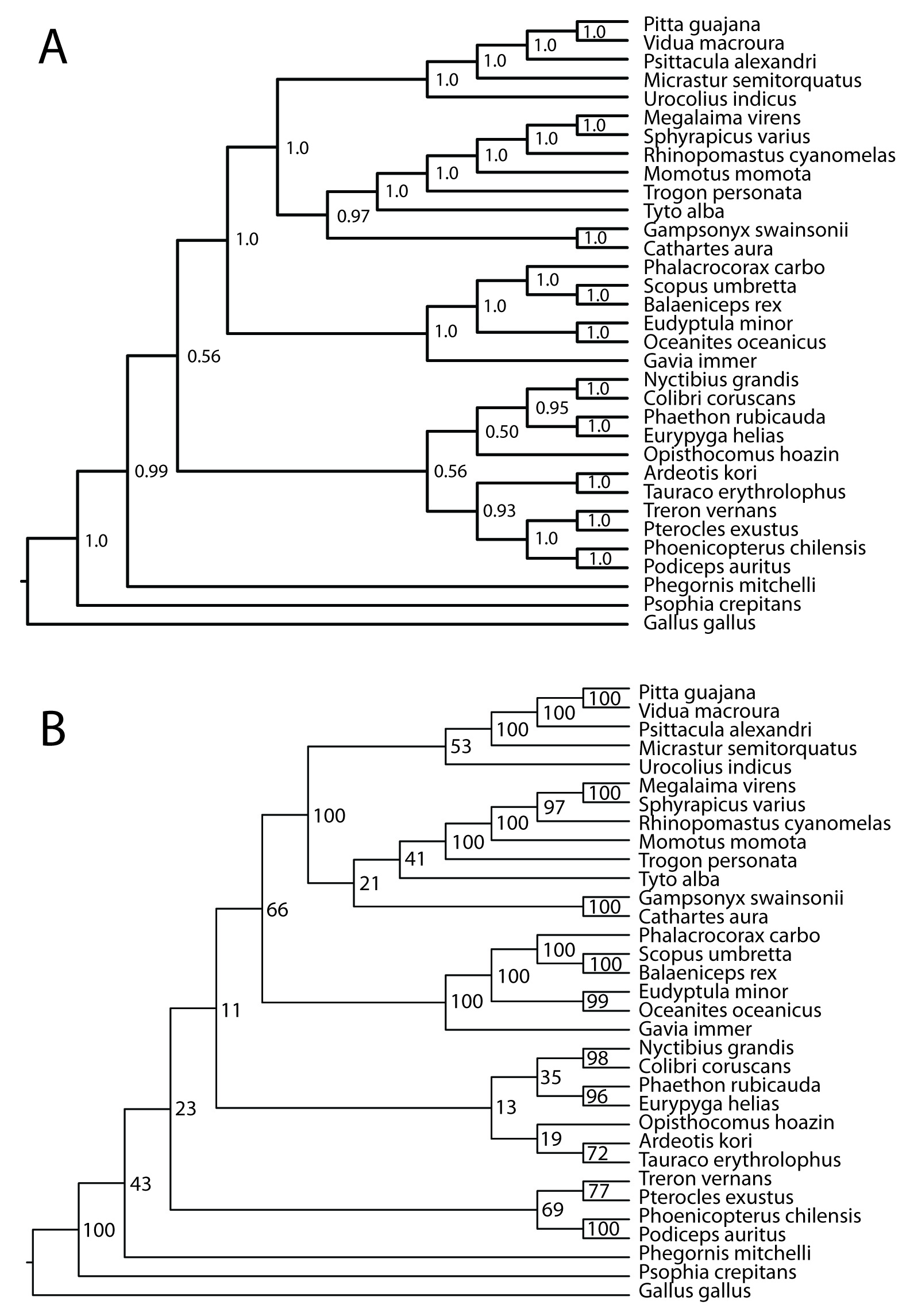
**Figure S1. Fully resolved trees from the 1,541 locus analysis with support values.** **A.** Bayesian tree. **B.** maximum-likelihood tree.
