# Supplementary figures and images for: A Phylogeny of Birds Based on Over 1,500 Loci Collected by Target Enrichment and High-Throughput Sequencing
Source: PLoS One. 2013 Jan 29;8(1):e54848. doi: 10.1371/journal.pone.0054848 (PMC3558522; doi:10.1371/journal.pone.0054848)

**Figure S2. Phylogram of the 1,541 locus Bayesian tree.**


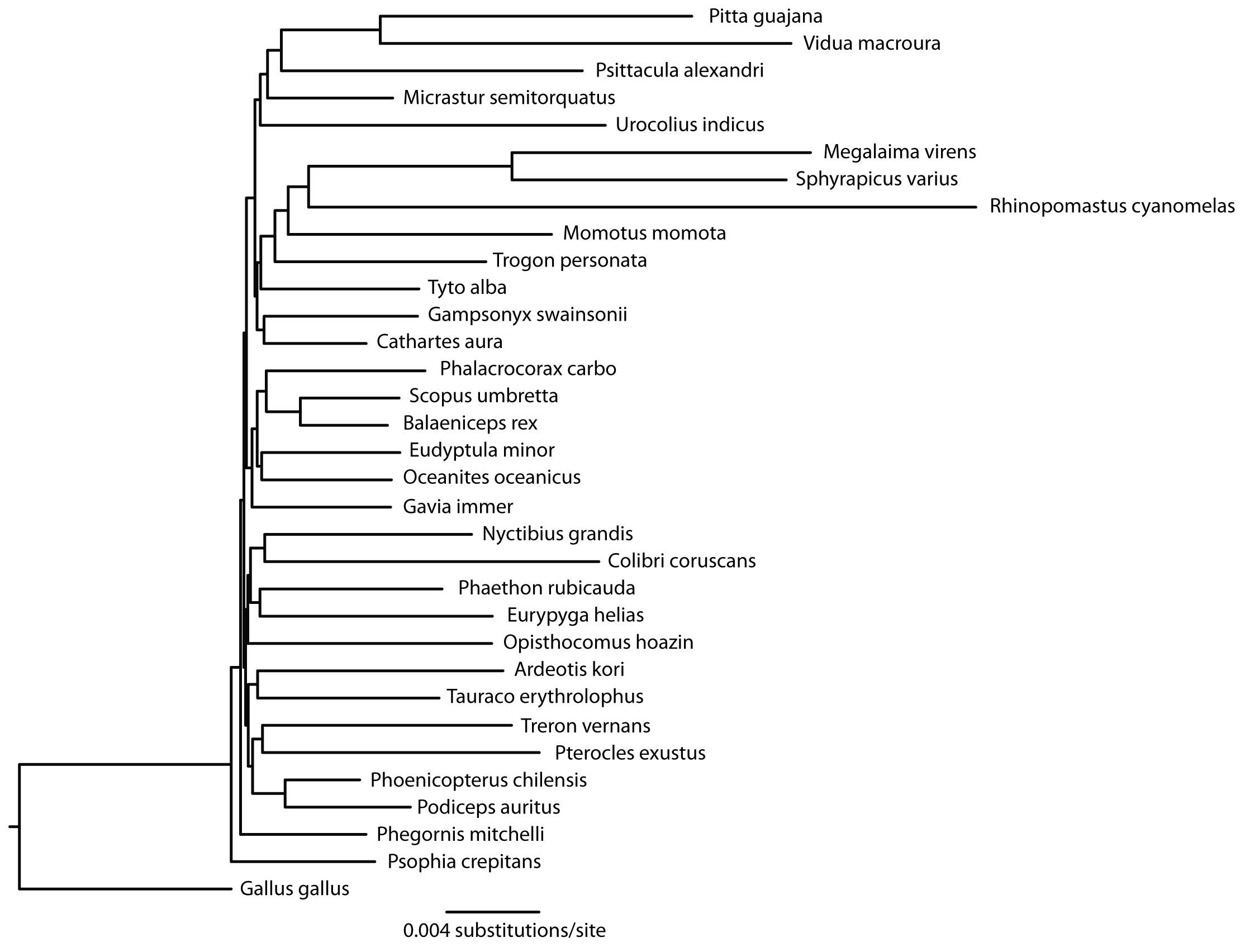

Supplement: Figure S2 — Phylogram of the 1,541 locus Bayesian tree. (DOCX) [file pone.0054848.s002.docx]
