# Supplementary material for: A Phylogeny of Birds Based on Over 1,500 Loci Collected by Target Enrichment and High-Throughput Sequencing
Source: PLoS One. 2013 Jan 29;8(1):e54848. doi: 10.1371/journal.pone.0054848 (PMC3558522; doi:10.1371/journal.pone.0054848)
Supplement: Table S1 — Indels greater than 1 bp. Informative indels (n = 13) that corroborate Bayesian phylogeny are indicated with bold names. (DOCX) [file pone.0054848.s004.docx]

**Table S1. Indels greater than 1 bp. Informative indels (n=13) that corroborate Bayesian phylogeny are indicated with bold names.**

| **UCE^1^** | **size^2^** | **type^3^** | **Species (informative indels in bold)** |
| --- | --- | --- | --- |
| chr8_4091 | 2 | deletion | *Rhinopomastus, Sphyrapicus* |
| chr1_32309 | 3 | insertion | *Pitta, Rhinopomastus, Psittacula, Momotus, Podiceps, Gampsonyx, Tyto, Pterocles, Colibri, Sphyrapicus, Nyctibius, Cathartes, Phoenicopterus, Eurypyga, Megalaima, Urocolius, Gavia, Treron* |
| chr3_5661 | 2 | insertion | *Rhinopomastus, Sphyrapicus* |
| chr3_5661 | 3 | deletion | *Eurypyga, Opisthocomus* |
| chr13_707 | 6 | deletion | *Eurypyga, Treron* |
| chr9_3551 | 4 | deletion | *Colibri, Rhinopomastus, Treron, Eurypyga* |
| chr9_3551 | 7 | deletion | ***Megalaima, Sphyrapicus*** |
| chr9_3551 | 3 | deletion | *Psittacula, Ardeotis* |
| chr2_21162 | 4 | deletion | *Opisthocomus, Treron, Phoenicopterus, Podiceps* |
| chr13_2902 | 3 | insertion | *Gampsonyx, Phalacrocorax* |
| chr7_6244 | 5 | insertion | *Balaeniceps, Phalacrocorax* |
| chr2_3317 | 4 | deletion | ***Scopus, Balaeniceps*** |
| chr15_3386 | 4 | deletion | *Psittacula, Gampsonyx* |
| chr15_3386 | 4 | deletion | *Urocolius, Scopus* |
| chr1_32247 | 4 | deletion | *Momotus, Urocolius* |
| chr1_32247 | 4 | deletion | ***Phoenicopterus, Podiceps*** |
| chr3_5522 | 10 | deletion | *Sphyrapicus, Phaethon* |
| chr5_10912 | 2 | deletion | ***Megalaima, Sphyrapicus*** |
| chr2_23600 | 5 | insertion | ***Megalaima, Sphyrapicus*** |
| chr7_10289 | 2 | deletion | *Momotus, Sphyrapicus* |
| chr8_5177 | 6 | deletion | *Megalaima, Urocolius* |
| chr1_32424 | 2 | deletion | *Colibri, Ardeotis* |
| chr6_4126 | 6 | insertion | *Colibri, Pterocles, Rhinopomastus, Gampsonyx, Podiceps, Psophia* |
| chr6_4126 | 4 | insertion | *Pitta, Gampsonyx* |
| chr12_1611 | 4 | deletion | ***Momotus, Sphyrapicus, Megalaima*** |
| chr2_12990 | 4 | deletion | ***Megalaima, Sphyrapicus*** |
| chr3_19997 | 2 | deletion | *Rhinopomastus, Urocolius, Psophia* |
| chr7_10443 | 3 | deletion | *Megalaima, Treron, Sphyrapicus* |
| chr8_4221 | 3 | deletion | *Rhinopomastus, Motmotus, Sphyrapicus* |
| chr1_15632 | 3 | deletion | *Sphyrapicus, Megalaima, Opisthocomus* |
| chr11_3419 | 3 | deletion | *Balaeniceps, Motmotus, Gampsonyx* |
| chr7_10549 | 4 | deletion | *Tauraco, Phalacrocorax* |
| chr15_2007 | 2 | deletion | *Sphyrapicus, Megalaima, Psittacula, Tauraco, Podiceps* |
| chr9_3633 | 6 | deletion | ***Scopus, Balaeniceps*** |
| chr2_18663 | 2 | deletion | *Rhinopomastus, Eurypyga* |
| chr6_8088 | 4 | deletion | *Nyctibius, Psittacula, Oceanites* |
| chr1_28710 | 3 | deletion | *Sphyrapicus, Eudyptyla* |
| chr1_28710 | 3 | deletion | ***Sphyrapicus, Megalaima*** |
| chr11_4777 | 3 | deletion | ***Phoenicopterus, Podiceps*** |
| chr5_14389 | 2 | deletion | ***Megalaima, Sphyrapicus*** |
| chr1_5427 | 2 | deletion | ***Balaeniceps, Scopus*** |
| chr5_2017 | 2 | deletion | ***Megalaima, Sphyrapicus*** |
| chr2_18589 | 2 | deletion | *Cathartes, Psophia* |
| chr2_18589 | 2 | deletion | *Rhinopomastus, Psittacula, Ardeotis* |

| 1 Location relative to chicken genome |
| --- |
| 2 in base pairs |
| 3 relative to chicken outgroup |
